# Propagation dynamics of *Anaplasma marginale* in a Brazilian tick cell line (RBME-6) derived from *Rhipicephalus microplus*

**DOI:** 10.1590/S1984-29612025075

**Published:** 2025-12-15

**Authors:** Gustavo Seron Sanches, Ana Carolina Castro-Santiago, Ricardo Bassini-Silva, Ana Cláudia Calchi, Simone Michaela Simons, Luís Antonio Mathias, Pablo Henrique Nunes, Rosangela Zacarias Machado, Marcos Rogério André, Darci Moraes Barros-Battesti

**Affiliations:** 1 Universidade Estadual Paulista – UNESP, Faculdade de Ciências Agrárias e Veterinárias – FCAV, Departamento de Patologia, Reprodução e Saúde Única, Vector-Borne Bioagents Laboratory – VBBL, Jaboticabal, SP, Brasil; 2 Universidade de São Paulo – USP, Faculdade de Medicina Veterinária e Zootecnia – FMVZ, Departamento de Medicina Veterinária Preventiva e Saúde Animal, São Paulo, SP, Brasil; 3 Instituto Butantan, Laboratório de Coleções Zoológicas, São Paulo, SP, Brasil; 4 Instituto Butantan, Laboratório de Parasitologia, São Paulo, SP, Brasil; 5 Universidade Estadual Paulista – UNESP, Faculdade de Ciências Agrárias e Veterinárias – FCAV, Departamento de Patologia, Reprodução e Saúde Única, Laboratório de Doenças Parasitárias, Jaboticabal, SP, Brasil; 6 Instituto Latino-Americano de Ciências da Vida e da Natureza, UNILA, Foz do Iguaçu, PR, Brasil

**Keywords:** Replication kinetics, Anaplasma marginale, tick cell line, RBME-6, Cinética de replicação, Anaplasma marginale, linhagem celular de carrapatos, RBME-6

## Abstract

*Anaplasma marginale* is an obligate intraerythrocytic alpha-proteobacterium that causes bovine anaplasmosis, which is responsible for economic losses in the livestock industry worldwide. Although the mechanism of *A. marginale* infection in tick cell lines has been extensively studied, replication dynamics have not been investigated so far. This study presents the replication kinetics of *A. marginale* in the RBME-6 tick cell line derived from *Rhipicephalus microplus* over time. Cell cultures were infected with corpuscles of *A. marginale* isolated from bovine blood. The replication curve was generated by the number of copies of the *msp1β* gene obtained by reverse-transcription quantitative PCR (RT-qPCR) using complementary DNA (cDNA) synthesized from RNA. An initial lag phase and an exponential replication phase were recorded. A subsequent death phase was observed, with rapidly decreasing numbers of *A. marginale msp1β* copies. The surviving population of *A. marginale* entered a long-term stationary phase from 13 dpi until 29 dpi. Light microscopy was used to monitor the infection in the cells. The propagation kinetics of *A. marginale* in RBME-6 cells were shown to be essential for guiding future studies aimed at unraveling the crosstalk between this bacterium and its biological tick vector.

## Resumo

*Anaplasma marginale* é uma alfa-proteobactéria intraeritrocítica obrigatória que causa anaplasmose bovina, responsável por perdas econômicas na indústria pecuária mundial. Embora o mecanismo de infecção por *A. marginale* em linhagens celulares de carrapatos tenha sido extensivamente estudado, a dinâmica de replicação não foi investigada até o momento. Este estudo apresenta a cinética de replicação de *A. marginale* na linhagem RBME-6 derivada de *Rhipicephalus microplus* ao longo do tempo. Culturas de células foram infectadas com corpúsculos de *A. marginale* isolado de sangue bovino. A curva de replicação foi gerada pelo número de cópias do gene *msp1β* obtidos por PCR quantitativa de transcrição reversa (RT-qPCR) por meio DNA complementar (cDNA) sintetizado a partir do RNA. Uma fase inicial e uma fase de replicação exponencial foram registradas. Uma fase subsequente de morte foi observada, com rápida diminuição do número de cópias de *msp1β* de *A. marginale*. A população sobrevivente de *A. marginale* entrou em fase estacionária de longo prazo de 13 dpi até 29 dpi. Microscopia de luz foi utilizada para monitorar a infecção nas células. A cinética de propagação de *A. marginale* nas células RBME-6 mostrou-se essencial para orientar estudos futuros visando desvendar o *crosstalk* entre esta bactéria e seu carrapato vetor biológico.

## Introduction

*Anaplasma marginale* (Rickettsiales: Anaplasmataceae) is an obligate intraerythrocytic alpha-proteobacterium that causes bovine anaplasmosis, which is responsible for significant economic losses in the livestock industry worldwide (Kocan et al., 2004; Cabezas-Cruz et al., 2019). The disease can be transmitted transplacentally during gestation, mechanically by fomites or blood-sucking flies and biologically by tick vectors during their blood meal (Kocan et al., 2010). About 20 tick species worldwide, mainly those belonging to the genera *Rhipicephalus* and *Dermacentor*, have been pointed as vectors of *A. marginale* (de la Fuente et al., 2007a). In Brazil, the cattle tick *Rhipicephalus microplus* (Canestrini, 1888) is considered its main vector (Passos, 2012). While erythrocytes represent the only known niche of *A. marginale* replication in cattle, the cycle in ticks seems to be more complex, with pathogen migration through various organs, from intestinal cells to salivary glands (Kocan et al., 2004; Cabezas-Cruz et al., 2016).

The main method for controlling anaplasmosis include arthropod control through chemical acaricides, treatment with antibiotics to prevent clinical anaplasmosis, and vaccination (Kocan et al., 2010). The use of chemical acaricides and antibiotic therapy has long-term disadvantages because they are expensive and contribute to selection of resistant ticks and bacteria, respectively (Byaruhanga et al., 2020). Vaccines are economically and ecologically favorable alternatives for the control of bovine anaplasmosis. However, although live and inactivated vaccines have been tested for over 20 years (Rodríguez et al., 2000; de la Fuente et al., 2001; Kocan et al., 2003), there is still no commercially effective vaccine available (Salinas-Estrella et al., 2022). Since then, many vaccine candidates have been evaluated, with significant advances, but challenges persist (de la Fuente et al., 2017; Salinas-Estrella et al., 2022).

The first tick cell line, derived from nymphal tissues of *Rhipicephalus appendiculatus* Neumann, 1901, was established in 1975 (Varma et al., 1975). Many tick cell cultures have been developed, most of them obtained from egg masses, and are widely used for isolation and propagation of several pathogens. The first *in vitro* continuous culture system for *A. marginale* was established at the end of the last century, using the IDE8 tick cell line from *Ixodes scapularis* Say, 1821 (Munderloh et al., 1996). Since then, different studies on tick-pathogen interactions, using tick cell lines as *in vitro* systems have reduced the use of cattle in research (Bell-Sakyi et al., 2007; 2018). Indeed, tick cell lines have also been used for the isolation and propagation of several economically important tick-borne pathogens (Baêta et al., 2015).

Propagation of *A. marginale* in cell lines derived from different tick species has been successfully established over the past three decades (Munderloh & Kurtti, 1989; Baêta et al., 2015; Husin et al., 2021). The infected cell lines were originated from the following tick species: *Dermacentor variabilis* Say, 1821; *Rhipicephalus sanguineus* (Latreille, 1806) (Hidalgo et al., 1989); *Dermacentor andersoni* Stiles, 1908; *I. scapularis* (Munderloh et al., 1994; 1996; Blouin et al., 1998; Zweygarth et al., 2006; Baêta et al., 2015); and *R. microplus* (Esteves et al., 2009). The mechanisms of *A. marginale* infection in tick cell lines have been well documented (Hidalgo et al., 1989; Munderloh et al., 1994; 1996; Blouin et al., 1998; Rodríguez et al., 2000; Kessler, 2001; de la Fuente et al., 2001; Kocan et al., 2004; Cabezas-Cruz et al., 2016; Hove et al., 2022), but studies on the replication kinetics of this pathogen in different tick cell lines are scarce. The IDE8 cell line, originating from *I. scapularis*, was used to propagate *A. marginale* for the first time, remaining infective to cattle after several passages in culture (Munderloh et al., 1996; Blouin & Kocan, 1998), in addition to isolating and propagating other strains of the bacterium (Blouin et al., 2002; Bastos et al., 2009; Baêta et al., 2015). In the ISE6 line, originating from the same tick species (*I. scapularis*), strains of *Anaplasma phagocytophilum* were successfully isolated and propagated (Woldehiwet et al., 2002; Silaghi et al., 2011). Furthermore, ISE6 cells have also been used to evaluate the effects of antibiotics on *A. marginale* (Alonso et al., 2020). These two cell lines (IDE8 and ISE6) played an essential role in studies of differential gene transcription and expression of outer membrane proteins, in the search for vaccine candidates against *A. marginale* (Brayton et al., 2006).

The present study aimed to evaluate the replication dynamics of *A. marginale* in the RBME-6, a tick cell line derived from a Brazilian strain of *R. microplus* in a closed system, without replacement of tick cells. Understanding the replication kinetics is essential to unravel the interaction between *A. marginale* and *R. microplus* cell lines, in addition to guiding future experimental infection studies using this biological model.

## Material and Methods

### RBME-6 tick cell line

*Mycoplasma*-free *R. microplus* (RBME-6) cell cultures (Lima-Duarte et al., 2021) were maintained in 25 cm^2^ culture flasks (Corning, NY, USA) containing 4 mL of Leibovitz culture medium (L15-B) (Vitrocell Embriolife, SP, Brazil) supplemented with 10% tryptose phosphate broth (TPB) (BD DIFCO, MD, USA), Lipoprotein (Gibco, ThermoFisher, MA, USA), Vitamins (Vitrocell Embriolife, SP, Brazil) and L-Glutamine (Vitrocell Embriolife, SP, Brazil), added to 20% heat-inactivated fetal bovine serum (FBS) (Vitrocell Embriolife, SP, Brazil). The flasks were stored in an incubator at 30 °C. The medium was replaced once a week.

### Infection of RBME-6 cells with Anaplasma marginale

*Anaplasma marginale* (Jaboticabal strain) was isolated from cryopreserved blood of a highly parasitized calf, from the municipality of Jaboticabal (Dall’Agnol et al., 2021), state of São Paulo, Brazil (21° 15′ 18″ S; 48° 19′ 20″ W), under authorization from the Animal Ethics Committee (No. 296/22). Infected blood was collected in tubes containing EDTA (BD Vacutainer, NJ, USA) from the splenectomized calf under aseptic conditions. The blood was aliquoted into 1.5 mL cryotubes blood vials (Corning, NY, USA), with addition of 5% Dimethyl Sulfoxide (DMSO) (Sigma-Aldrich, MO, USA), and was frozen in a -80 °C freezer for 24 hours, then transferred to liquid nitrogen at -196 °C until use.

Before infection, “MOPS medium” was prepared. For this purpose, pure L15-B medium (without supplements) was prepared and 10 mM organic buffer 3-(N-morpholino) propanesulfonic acid (MOPS) (Sigma-Aldrich, MO, USA), 0.25% NaHCO3 (Sigma-Aldrich, MO, USA) (Munderloh et al., 1996) supplemented with 5% iron-enriched FBS (HyClone, Fisher Scientific, ThermoFisher, MA, USA) were added. Separately, MOPS medium without serum (serum-free) was also prepared only for the following washing steps.

**Figure 1:**
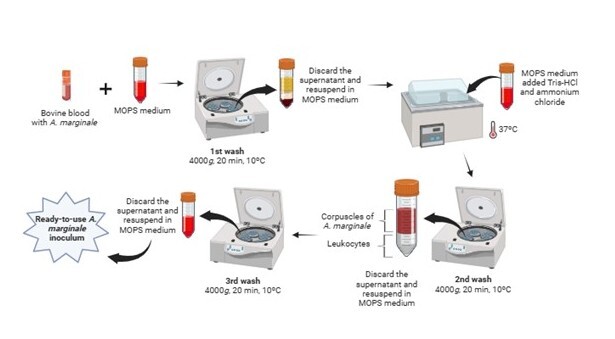
Preparation of *Anaplasma marginale* inoculum for infection into *Rhipicephalus* (*Boophilus*) *microplus* cell line (RBME-6).

A diagram shows how the *A. marginale* inoculum was prepared for infection (Figure 1). Briefly, 10 cryotubes were manually thawed and transferred to 50 mL Falcon tubes (KASVI, PR, Brazil). An aliquot of the blood was stored in a -20 °C freezer for molecular analyses. Then, MOPS serum-free medium was added to each tube and submitted to centrifugation for 20 minutes at 4000 x *g* at 10 °C (1st wash). The supernatant was carefully discarded, and the pellet was resuspended with MOPS serum-free medium. A tris-ammonium chloride solution (1 Tris-HCl: 9 Ammonium Chloride) was added and the tube was heated in a water bath at 37 °C for approximately 5 minutes. After resuspension, the tube was submitted to centrifugation under the same conditions described previously (2nd wash). The supernatant was discarded. Carefully, the pellet containing *A. marginale* corpuscles was transferred to a new Falcon tube and MOPS serum-free medium was added to complete the volume to 50 mL. Again, centrifugation was performed under the same conditions described above (3rd wash). The supernatant was carefully discarded, and the pellet was resuspended in approximately 5 mL of MOPS medium (with 5% iron-enriched FBS). Thus, the inoculum was now ready for infection.

For infection, a total of 60 flasks of cells from passages 29 and 30 (P29 and P30) contained normal culture medium (L15-B with FBS) removed completely and were infected with the inoculum containing *A. marginale* corpuscles, 2.5 mL of MOPS medium with 5% FBS enriched with iron totaling 3 mL in each flask. A MOI (Multiplicity of Infection) =1 was used for RBME-6 cells. The infected cells flasks were kept in an incubator at 34 °C until the day of collection. Cell adhesion was observed using an inverted microscope (Eclipse TS100, Nikon, Tokyo, Japan) daily. For the control group, the same amount of cell flasks non-infected were maintained in MOPS medium with 5% FBS enriched with iron and kept at 34 °C. Twenty-four hours post-inoculation, over a period of 29 days post-infection (dpi), two infected flasks were removed from the experiment and submitted to RNA extraction. For the flasks that continued in the trial, the culture medium was completely changed once a week, but without RBME-6 cells added. For non-adherent cells, 3 mL of MOPS medium was collected, and the content was transferred to identified 1.5 mL. Then, 3 mL of MOPS medium was added, and the adherent cells were scraped (Corning, NY, USA) and transferred to identified 1.5 mL Eppendorf microcentrifuge tubes. After all samples were collected, they were centrifuged at 12,000 RPM for 5 minutes, and after discarding the supernatant, the pellet was resuspended in 200 µL of RNAlater (Sigma-Aldrich, MO, USA).

### Extraction of DNA, RNA and cDNA synthesis

DNA was extracted from an aliquot of the blood using the DNeasy Blood & Tissue Kit (Qiagen, Hilden, Germany), while total RNA from adherent and non-adherent cells was extracted using the ReliaPrep RNA Mini Cell Prep System (Promega, WI, USA), both following the manufacturer's recommendations. One microliter of RNA extracted from each sample was aliquoted for complementary DNA (cDNA) synthesis using the GoScript Reverse Transcription System (Promega, WI, USA), according to the manufacturer's instructions. The concentration and purity of DNA and RNA were estimated using the spectrophotometer Nanodrop 2000c (ThermoFisher, MA, USA).

### Quantitation of Anaplasma marginale msp1β gene and replication curve

The number of copies/µL obtained from the amplification of 95 bp of the "*major surface protein*" (*msp1β*) gene of *A. marginale* (Carelli et al., 2007) was quantified using a reverse-transcription quantitative PCR (RT-qPCR) assay (Table 1). The RT-qPCR was performed on synthesized cDNA samples synthesized from RNA to confirm bacterial survival and replication through absolute quantification of the number of copies/µL of the target fragment.

**Table 1:** Oligonucleotide primers and hydrolysis probe sequences for target gene used in qPCR assay in RBME-6 cells.

Agent	Target gene	Fragment size	Primer sequences (5'-3')	Reference
*Anaplasma marginale*	*msp1β*	95 bp	AM-F (TTGGCAAGGCAGCAGCTT)	Carelli et al. (2007)
			AM-R (TTCCGCGAGCATGTTGCAT)	
			AM-probe (6FAM-TCGGTCTAACATCTCCAGGCTTTCAT-BHQ1)	

The amplification reactions were carried out in duplicates in 96-well plates (Bio-Rad, CA, USA). Amplification reactions were performed using a final volume of 10 µL, containing a mixture of 1 µL of extracted cDNA, 0.2 μM of primers, 5 µL of PCR buffer (GoTaq Probe qPCR Master Mix, Promega, WI, USA) and 0.2 µL of hydrolysis probe (Integrated DNA Technologies, IA, USA), 5 µL of PCR buffer (GoTaq qPCR Master Mix, Promega, WI, USA) and sterilized ultrapure water (Nuclease-Free Water, Promega, WI, USA) q.s.p. 10 µL. Sterile ultrapure water was used as a negative control for the reaction. The cycles were performed under the following conditions: 95ºC for 10 minutes and 40 cycles of 15 seconds at 95ºC and 1 minute at 60ºC. The amplification reactions were conducted in a CFX96 Thermal Cycler (Bio-Rad, CA, USA). The reaction efficiency was calculated by serial dilutions made to construct a standard curve with different concentrations of plasmid DNA containing the target sequence (2.0 x 10^7^ copies/µL to 2.0 x 10^0^ copies/µL). The number of plasmid copies was determined according to the formula (Xg/µL DNA/ [plasmid size (bp) x 660]) x 6,022 x 10^23^ x plasmid copies/µL. The replication curve of *A. marginale* in RBME-6 cells was constructed based on the number of *msp1β* gene copies of the pathogen obtained from the samples.

### Light microscopy of RBME-6 infected cells

Replication rate of *A. marginale* infection in RBME-6 cells was monitored by examination of Giemsa-stained cytocentrifuge smears prepared at the time-points of 1, 2, 3, 5, 11 and 15 dpi. Briefly, 100 µL of the suspension containing adherent cells were centrifuged for 5 min at 1000 × *g* (Cytospin 4, ThermoFisher, MA, USA). The smears were fixed in methanol and Giemsa-stained. The number of parasitized cells (i.e. presenting *A. marginale* morulae or inclusion corpuscles) was counted, using a light microscope BX53 (Olympus, Tokyo, Japan) coupled to a digital camera DP73 (Olympus, Tokyo, Japan).

### Transmission electron microscopy (TEM) of RBME-6 cells

Adherent uninfected and *A. marginale*-infected RBME-6 cells were collected at 3, 5, 7 and 11 dpi. The tick cells were fixed in Karnovsky’s solution for 2 h at 4 °C and subsequently post-fixed in 1% osmium tetroxide solution in the same buffer for 1 h at 4 °C. Dehydration was carried out using a graded ethanol series (70-100%). Following dehydration, the samples were infiltrated with a 1:1 mixture of propylene oxide with Epon resin for 2–3 h. The cell suspensions were transferred to a BEEM capsule containing pure resin and centrifuged at 900 x *g* for 5 min. After replacing the resin, the samples were centrifuged again, and the capsules containing the cell pellets were polymerized at 60 °C for 2–3 days. Ultrathin sections (60–70 nm) were obtained, mounted on copper grids, and stained with 2% aqueous uranyl acetate for 10 min, followed by lead citrate for 3–5 min. The cells were then examined under a transmission electron microscope (LEO 906E, Zeiss, Oberkochen, Germany). Images were captured with a CCD camera (Mega View III, Olympus, Tokyo, Japan) and processed with iTEM software (Universal TEM Imaging Platform, Olympus Soft Imaging Solutions GmbH) at the Electron Microscopy Laboratory of the Institute of Biosciences, São Paulo State University (UNESP, Rio Claro, SP, Brazil).

### Statistical analysis

To estimate the numbers of *A. marginale msp1β* gene copies in RBME-6 adherent and non-adherent cells, statistical analyses were performed considering four biological replicates for each time point. Prais-Wisten regression analysis was conducted following the model of Antunes & Waldman (2002), using the statistical software R, to examine the temporal trend. The angular coefficient and its standard error were used to calculate the rate variation and the 95% confidence interval using the t-distribution multiplier. The adherent cells were compared with the number of the non-adherent cells. A comparison was conducted using nonparametric analysis for longitudinal data (Brunner & Langer, 2000). The analysis was performed in R using the nparLD package.

To avoid the problem of multiple comparisons, these analyses were replaced by nonparametric ANOVA for repeated measures, which allows for all comparisons of both factors (treatments and time points) to be made simultaneously, in addition to circumventing the problem of the lack of Gaussian distribution in some of the data.

## Results

### Quantitation and replication curve of Anaplasma marginale in RBME-6 cells

**Figure 2:**
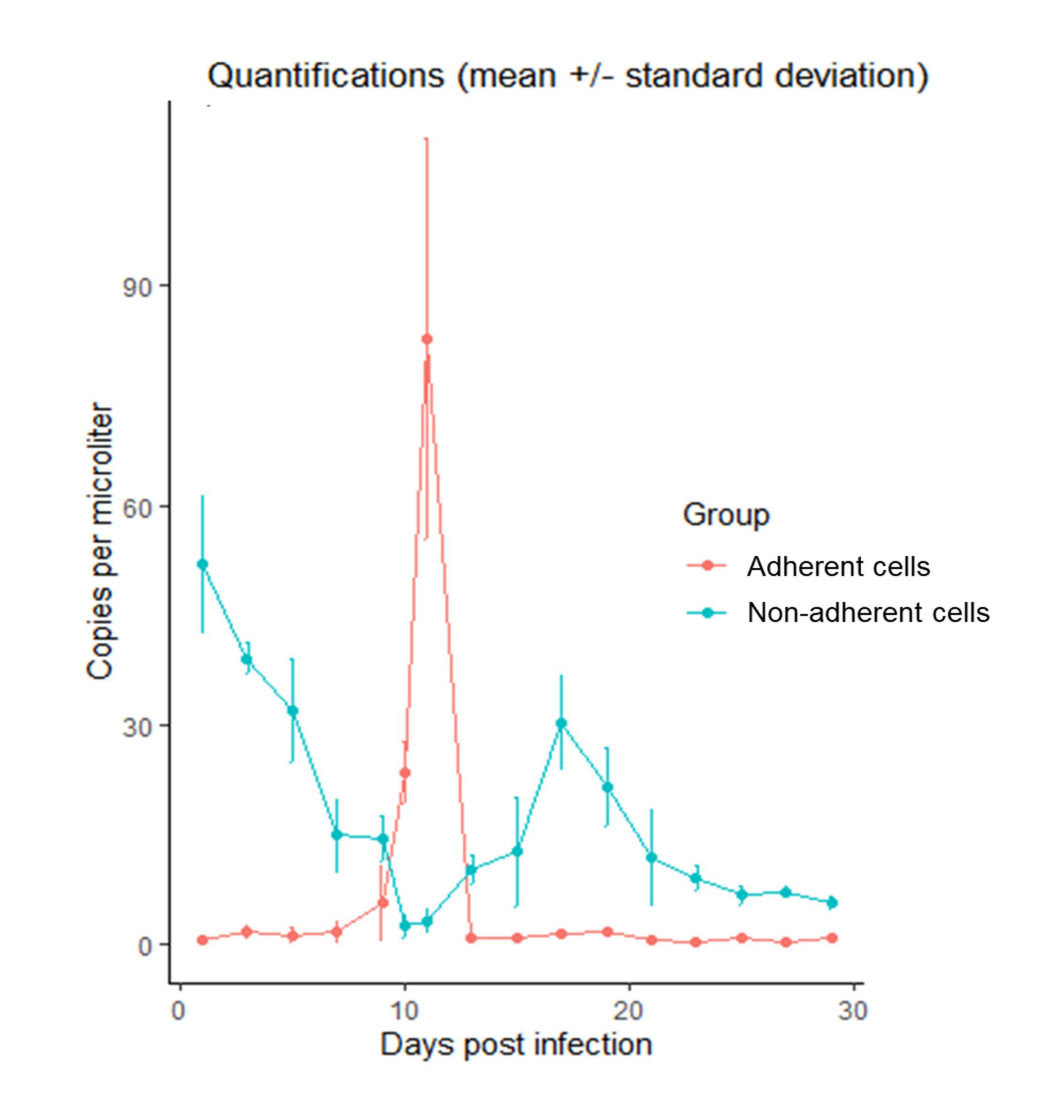
Replication curve of *Anaplasma marginale* in RBME-6 cells estimated by absolute quantifications obtained by qPCR. Lag phase (1–7 dpi), exponential phase (7–11 dpi), death phase (11–13 dpi) and a long-term stationary phase (13–29 dpi).

The estimated quantification of *A. marginale* present in the inoculum was 2.60 x 10^3^ copies/µL. The efficiency, R2, slope and y-axis intercept of the reaction were 104.2%, 0.991, 3.243 and 32.988, respectively. Preliminary tests showed that when inoculated 130 µL (~ 3.38 x 10^3^ copies) in 3 mL of MOPS medium with 5% FBS enriched with iron, RBME-6 cells in monolayer remained adhered to the bottom of the flask. On the other hand, when inoculated with 250 µL of inoculum (~ 6.5 x 10^3^ copies), the cells adhered to the bottom of the flask detached within 2 hours. For this reason, the subsequent experimental infection was performed using the first amount of *A. marginale*. In adherent cells, the replication curve demonstrated that there was an initial lag phase (1–7 dpi). This phase was followed by an exponential replication phase (7–11 dpi). On 11 dpi, the highest mean number of copies was recorded as 82.82 ± 27.49 copies/µL. Figure 2 shows the replication curve of *A. marginale* in RBME-6 cell line. The temporal trend analysis for the quantity DNA copies of *A. marginale* in adherent cells showed an increase of 108.21% from the lag phase to the log phase (95% CI: 23.85 to 250.03; p = 0.013). The mean generation time for *A. marginale* in these cells was 0.69 days (or 16.56 hours). A subsequent rapid decrease in DNA copies of *A. marginale* quantification was observed (11–13 dpi), thus characterizing the death phase. After 13 dpi, the bacteria entered a long-term stationary phase, such that the number of pathogens remained low until 29 dpi. The inverse was observed in the quantifications of *A. marginale* in non-adherent cells when 24 h post-infection, the number of copies reached its maximum (51.83 ± 9.33 copies/µL), while the number of DNA copies of *A. marginale* in RBME-6 monolayer cells was 0.48 ± 0.14 copies/µL. The temporal trend analysis for non-adherent cells showed a decrease of 42.39% in the quantity of *A. marginale* from day 1 to day 13 (95% CI: -57.54 to –21.85; p = 0.004), and of 21.28% from day 15 to 29 (95% CI: -31.42 to -9.65; p = 0.005). The estimated quantification of *A. marginale* showed statistically significant differences (p < 0.05) between the number of copies in non-adherent and adherent cells.

### Light microscopy of RBME-6 infected cells

**Figure 3:**
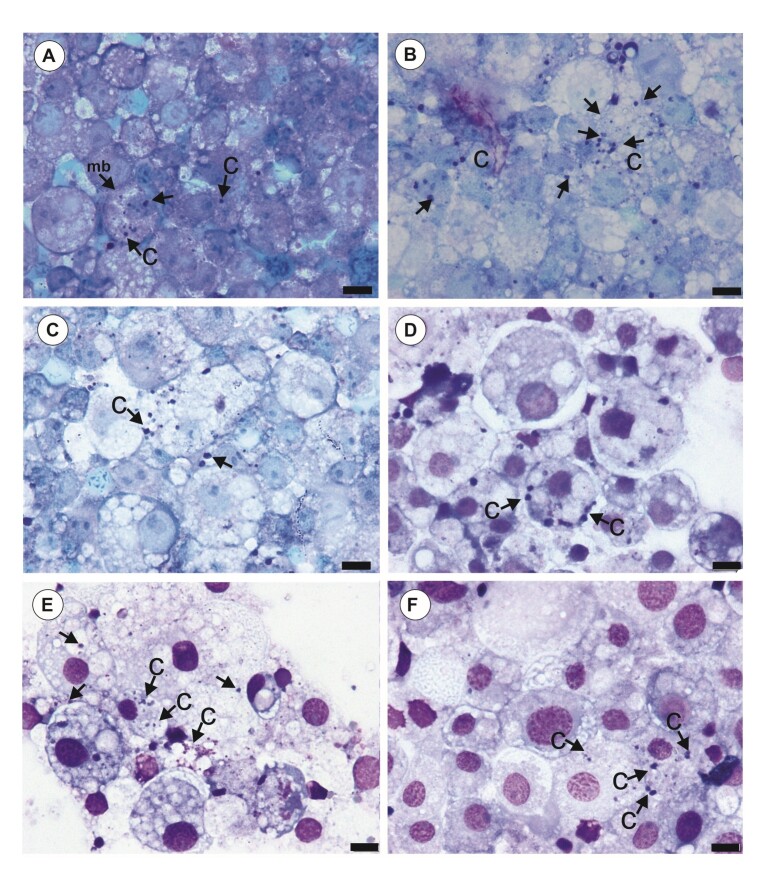
Replication of *Anaplasma marginale* in the RBME-6 cells monitored through examination of Giemsa-stained cytocentrifuge smears: (A) 1 day post infection (dpi); (B) 2 dpi; (C) 3 dpi; (D) 5 dpi; (E) 11 dpi; and (F) 15 dpi. The presence of corpuscles of *A. marginale* forming colonies (small black points) is represented by the black arrow. Abbreviations: C, corpuscles of *Anaplasma*; mb, RBME-6 cell membrane. Scale bars: 10 µm.

The number of *A. marginale* corpuscles in adherent and non-adherent cells of the RBME-6 line during the 29-day period is shown in Figure 3. The presence of *A. marginale* colonies (morulae) was observed on day 1 post-infection (Figure 3A) in the cytoplasm of some cells. On day 2 (Figure 3B) and day 3 post-infection (Figure 3C) there was an increase in the number of infected cells and colonies in the cytoplasm and around the nuclear region were observed. At 5 dpi (Figure 3D), large colonies at the periphery of the cytoplasm were observed, and at 11 dpi (Figure 3E) a large amount of *A. marginale* was observed in cells which presented vacuolated cytoplasm. Once most cells had been infected, they entered the death phase (apoptosis) at 11 dpi, with collapse of the cytoskeleton, loss of cellular contours, disassembly of the nuclear envelope and fragmentation of nuclear DNA. Between 15 dpi (Figure 3F) and 29 dpi, only a few infected cells were observed, and the remaining uninfected cells seemed to multiply again.

### Transmission electron microscopy of RBME-6 cells

**Figure 4:**
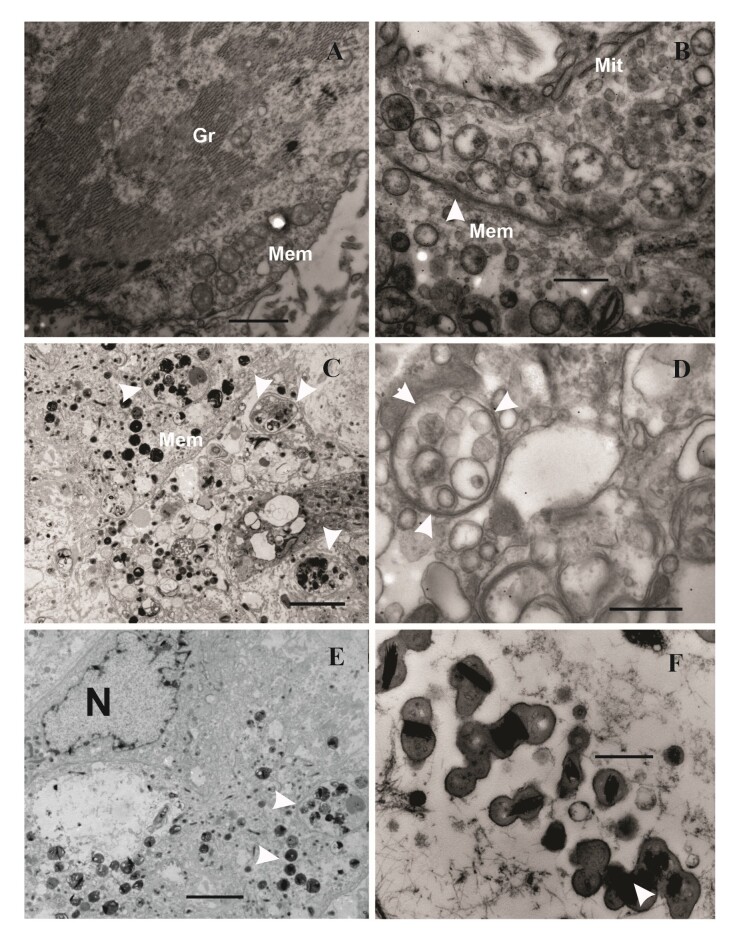
Transmission electron microscopy (TEM) images of the RBME-6 cells, non-infected and infected with *A. marginale* according to the day post infection (dpi). A-B: Cells from control group (non-infected), longitudinal and transverse cuts, respectively, with mitochondria and Golgi reticulum. C: RBME-6 cells 3 dpi with morulae formed vesicles (arrows), there are two membranes evolving the *A. marginale* corpuscules. D-E: Infected cells 5 dpi and 7 dpi filled with *A. marginale* corpuscles inside and out vesicles, respectively (arrows). F: Binary fission of bacteria 11 dpi. Abbreviations: Mem, membrane; Mit, mitochondria; N, nucleus; Gr, golgi reticulum. Scale-bars: A, C, E: 100 nm; B, D: 500 nm; F: 1000 nm.

Transmission electron microscopy (TEM) of non-infected and infected RBME-6 cells are shown in Figure 4. Once non-infected cells were inoculated (Figures 4A, 4B), the bacteria invade the cells and begin to multiply 3 dpi and produce colonies within the membranes (morulae) (Figure 4C). At 5 and 7 dpi the infected cells were filled with *A. marginale* corpuscles (Figure 4D, 4E). When infected after 11 days (Figure 4F), binary division of numerous cells was observed, with the appearance of electron-dense forms.

## Discussion

Microbial replication dynamics is a fundamental aspect of modern microbiology and biotechnology, since it is crucial for understanding host-pathogen interactions. This knowledge is particularly important for studying *A. marginale* infection in the *R. microplus* RBME-6 tick cell line, providing a basis for future studies using this biological model. To our knowledge, this is the first study to monitor the replication kinetics of *A. marginale* in RBME-6 cells over a 29-day period.

The replication curve was constructed by estimating the bacterial load using the number of copies/µL of the target fragment (*msp1β* gene) of *A. marginale* in adherent cells using a RT-qPCR assay. This technique was based on bacterial cDNA synthesized from RNA, providing an absolute quantification of viable pathogens compared to DNA quantification, avoiding overestimation caused by extracellular or degraded DNA, as bacterial transcripts degrade rapidly after cell death (Richards et al., 2008). It is important to emphasize that this gene was used because it is commonly used in the laboratory. However, interpretation of *msp1β* transcript abundance should be cautious, as transcript levels from a single gene are not necessarily proportional to the number of pathogenic cells. Gene expression can vary independently of replication due to transcriptional regulation, environmental conditions, or metabolic state, and RNA stability is not constant. In parallel, transcriptome analysis of RBME-6 cells infected with *A. marginale* was performed during the period of the present study, enabling characterization of the transcriptional response of the cells to the pathogen (unpublished data). The results indicated that significant differential expression of genes occurred, associated with bacterial replication and persistence, thus providing evidence of progression of the infection over time. Changes in the expression of genes related to the immune response, cellular metabolism and signaling processes were also observed, suggesting that the host cells were adapting to the presence of the pathogen. These findings confirm the susceptibility of the RBME-6 lineage to infection by the bacteria and provide insights into the molecular mechanisms involved in the tick-pathogen interaction.

Under optimal conditions, bacterial replication typically progresses through five phases: lag, exponential (log), stationary, death, and long-term stationary phases (Finkel, 2006; Rolfe et al., 2012). In our study, the replication of *A. marginale* in RBME-6 cells exhibited four distinct phases: a lag phase (1-7 dpi), an exponential replication phase (7-11 dpi), a rapid death phase (11-13 dpi), and a long-term stationary phase (13-29 dpi). During the lag phase, bacteria adapt to the replication environment, while the exponential phase is characterized by constant cell division driven by DNA replication, transcription, and translation (Madigan et al., 2019). Variations in replication kinetics are influenced by medium, temperature, and host cell species (Klumpp et al., 2009; Barros-Battesti et al., 2018).

Munderloh et al. (1996) reported replication of *A. marginale* in an IDE8 tick cells 34 days after infection, with infection rates increasing over successive passages. In contrast, RBME-6 cells showed rapid infection, with *A. marginale* colonies (morulae) visible as early as 1 dpi. By 3 dpi, ~50% of cells were infected, and electron-dense forms –indicative of infectious stage (Kocan et al., 1992) – were present.

Considering the global diversity of *A. marginale* strains, which differ in morphology, virulence, and transmission dynamics (Bastos et al., 2009; Passos, 2012; Baêta et al., 2015), future studies should focus on the infection dynamics of different strains in tick cell lines. The IDE8 and ISE6 cell lines derived from *I. scapularis* have supported the replication of multiple strains of *A. marginale* and other pathogens (Munderloh et al., 1999; Zweygarth et al., 2006). Munderloh et al. (2004) found that an *A. marginale* strain obtained from endothelial cells, when propagated in ISE6 cells, caused cell apoptosis and pathogen release into the supernatant after one week of infection. These same cells expressed heat shock proteins (HSPs) when infected with *Anaplasma* spp., pointing to the possibility of post-transcriptional mechanisms induced by the bacterium to control the tick response to infection (Villar et al., 2010). When infected with *A. phagocytophilum*, an inhibition of genes involved in the modulation of apoptosis pathways in ISE6 cells was observed, suggesting the use of bacterial mechanisms to facilitate cell infection (Alberdi et al., 2016). This hypothesis was confirmed when Villar et al. (2015) and Cabezas-Cruz et al. (2017a, b) verified, through transcriptomic, proteomic and metabolomic analyses, that this pathogen affects amino acid and carbohydrate pathways of infected ISE6 cells. They suggested that *A. phagocytophilum* alters these pathways, facilitating its infection and replication in tick cells. Kalil et al. (2017) observed that in *A. marginale*-infected IDE8 cells, the immune-associated metabolism of these cells can be manipulated by the pathogen to escape the deleterious effect of the oxidant-based immune response. Additionally, the in vitro culture technique of these *A. marginale*-infected cells has been used in functional research, discovering genes and proteins that exhibit distinct expression patterns within tick cells after infection (de la Fuente et al., 2007b; Kocan et al., 2009).

The first propagation of a Brazilian isolate of *A. marginale* (strain UFMG1) in *R. microplus* BME26 cells demonstrated slow infection dynamics, with intracellular colonies forming in the second passage (Esteves et al., 2009). In contrast, our study using RBME-6 cells revealed rapid infection dynamics without cell replacement, highlighting the need for further research on strain-specific interactions in tick cells.

Nutrient-rich media plays a crucial role in bacterial replication. Transition metals such as iron are essential for cellular functions, as 30% of bacterial proteins depend on metal cofactors (Zhang & Zheng, 2020). Iron supplementation has been shown to support the replication of *A. marginale* in *D. andersoni* tick cell lines (Solyman et al., 2022) and other rickettsial pathogens (Barros-Battesti et al., 2018). However, prolonged exposure to high iron concentrations can damage tick cell lines. To balance cell growth and viability, we used MOPS medium supplemented with 5% iron-enriched FBS for *A. marginale* inoculation.

While the average generation time for *A. marginale* in RBME-6 cells under our experimental conditions was 0.69 day (16.5 hours), 0.9 days were reported for *Rickettsia raoultii* in IDE8 cells (Husin et al., 2021). Notably, the stationary phase was absent in the experiment due to RBME-6 cells depletion, as it was decided not to add new cells to the infected flasks. This decision allowed to observe the behavior of *A. marginale* in the adherent and non-adherent cells. However, the medium was changed weekly to ensure the bacteria did not lack nutrients. This observation highlights the obligate intracellular nature of *A. marginale*, which relies on host cells for replication.

Light and electron microscopy provided insights into the dynamics of *A. marginale* infection in RBME-6 cells. At 2 dpi, infected cells exhibited colonies near the nuclear region, with large colonies observed at 5 dpi. During the exponential phase, intracellular pathogens, such as *Rickettsia rickettsii,* manipulate host cell pathways (e.g., NF-kB) to delay cell death and support replication (Clifton et al., 1998). At 11 dpi, extensive cytoplasmic vacuolization and cell lysis were evident, correlating with bacterial dissemination and the onset of the death phase.

## Conclusions

Our results describe the replication dynamics of *A. marginale* (Jaboticabal strain) in RBME-6 cells, estimated from the number of copies/µL for the *msp1β* gene based on absolute quantification of cDNA using a reverse-transcription quantitative PCR (RT-qPCR) assay, and emphasize the critical role of cell line selection and experimental conditions on understanding tick-pathogen interactions. Although it used mRNA, it was possible to conclude that there was bacterial replication. These findings reinforce the importance of complementary analyses for evaluating *A. marginale* multiplication in cellular models. The RBME-6 cell line, maintained in MOPS medium supplemented with 5% iron-enriched FBS at 34 °C, supported infection and propagation of *A. marginale* for at least 29 consecutive days without RBME-6 cells or pathogen replacement. Under these conditions, the replication curve exhibited four distinct phases: a lag phase (1–7 dpi), an exponential replication phase (7–11 dpi), reaching the highest mean copy number, a rapid death phase (11–13 dpi), and a long-term stationary phase (13–29 dpi). The absence of a stationary phase immediately following the exponential phase in this closed system suggests that nutrient availability alone is insufficient to sustain bacterial replication without RBME-6 cells replacement. This study provides a robust *in vitro* model to investigate interactions between *A. marginale* cells and ticks and serves as a valuable framework for future experimental research on rickettsial infections.

## References

[B001] Alberdi P, Espinosa PJ, Cabezas-Cruz A, de la Fuente J (2016). *Anaplasma phagocytophilum* manipulates host cell apoptosis by different mechanisms to establish infection. Vet Sci.

[B002] Alonso BI, Ventura ES, Esteves E, Galletti MFBM, Dall’Agnol B, Martins JR (2020). A tick cell line as powerful tool to screen the antimicrobial susceptibility of the tick-borne pathogen *Anaplasma marginale.*. Exp Parasitol.

[B003] Antunes JLF, Waldman EA (2002). Trends and spatial distribution of deaths of children aged 12-60 months in São Paulo, Brazil, 1980-98. Bull World Health Organ.

[B004] Baêta BA, Ribeiro CCDU, Teixeira RC, Cabezas-Cruz A, Passos LM, Zweygarth E (2015). Characterization of two strains of *Anaplasma marginale* isolated from cattle in Rio de Janeiro, Brazil, after propagation in tick cell culture. Ticks Tick Borne Dis.

[B005] Barros-Battesti DM, Machado RZ, André MR, de Sousa KCM, Franze DA, Lima-Duarte L (2018). Successful infection of tick cell cultures of *Rhipicephalus sanguineus* (tropical lineage) with *Ehrlichia canis.*. Vector Borne Zoonotic Dis.

[B006] Bastos CV, Passos LM, Vasconcelos MM, Ribeiro MFB (2009). *In vitro* establishment and propagation of a Brazilian strain of *Anaplasma marginale* with appendage in IDE8 (*Ixodes scapularis*) cells. Braz J Microbiol.

[B007] Bell-Sakyi L, Darby A, Baylis M, Makepeace BL (2018). The tick cell Biobank: A global resource for *in vitro* research on ticks, other arthropods and the pathogens they transmit. Ticks Tick Borne Dis.

[B008] Bell-Sakyi L, Zweygarth E, Blouin EF, Gould EA, Jongejan F (2007). Tick cell lines: tools for tick and tick-borne disease research. Trends Parasitol.

[B009] Blouin EF, de la Fuente J, Garcia-Garcia JC, Sauer JR, Saliki JT, Kocan KM (2002). Applications of a cell culture system for studying the interaction of *Anaplasma marginale* with tick cells. Anim Health Res Rev.

[B010] Blouin EF, Kocan KM (1998). Morphology and development of *Anaplasma marginale* (Rickettsiales: Anaplasmataceae) in cultured *Ixodes scapularis* (Acari: Ixodidae) cells. J Med Entomol.

[B011] Blouin EF, Saliki JT, Kocan KM, Rodgers SJ (1998). Evaluation of *Anaplasma marginale* from tick cell culture as an immunogen for cattle. Ann N Y Acad Sci.

[B012] Brayton KA, Palmer GH, Brown WC (2006). Genomic and proteomic approaches to vaccine candidate identification for *Anaplasma marginale.*. Expert Rev Vaccines.

[B013] Brunner E, Langer F (2000). Nonparametric analysis of ordered categorical data in designs with longitudinal observations and small sample sizes. Biom J.

[B014] Byaruhanga J, Odua F, Ssebunya Y, Aketch O, Tayebwa DS, Rwego IB (2020). Comparison of tick control and antibiotic use practices at farm level in regions of high and low acaricide resistance in Uganda. Vet Med Int.

[B015] Cabezas-Cruz A, Alberdi P, Valdés JJ, Villar M, de la Fuente J (2017). Anaplasma phagocytophilum infection subverts carbohydrate metabolic pathways in the tick vector, Ixodes scapularis.. Front Cell Infect Microbiol.

[B016] Cabezas-Cruz A, Espinosa PJ, Alberdi P, de la Fuente J (2019). Tick–pathogen interactions: the metabolic perspective. Trends Parasitol.

[B017] Cabezas-Cruz A, Espinosa PJ, Obregón DA, Alberdi P, de la Fuente J (2017). *Ixodes scapularis* tick cells control *Anaplasma phagocytophilum* infection by increasing the synthesis of phosphoenolpyruvate from tyrosine. Front Cell Infect Microbiol.

[B018] Cabezas-Cruz A, Zweygarth E, Vancová M, Broniszewska M, Grubhoffer L, Passos LMF (2016). Ehrlichia minasensis sp. nov., isolated from the tick Rhipicephalus microplus.. Int J Syst Evol Microbiol.

[B019] Carelli G, Decaro N, Lorusso A, Elia G, Lorusso E, Mari V (2007). Detection and quantification of *Anaplasma marginale* DNA in blood samples of cattle by real-time PCR. Vet Microbiol.

[B020] Clifton DR, Goss RA, Sahni SK, van Antwerp D, Baggs RB, Marder VJ (1998). NF-kappa B-dependent inhibition of apoptosis is essential for host cell survival during *Rickettsia rickettsii* infection. Proc Natl Acad Sci USA.

[B021] Dall’Agnol B, Webster A, Souza UA, Barbieri A, Mayer FQ, Cardoso GA (2021). Genomic analysis on Brazilian strains of *Anaplasma marginale.*. Rev Bras Parasitol Vet.

[B022] de la Fuente J, Contreras M, Estrada-Peña A, Cabezas-Cruz A (2017). Targeting a global health problem: vaccine design and challenges for the control of tick-borne diseases. Vaccine.

[B023] de la Fuente J, Garcia-Garcia JC, Blouin EF, Kocan KM (2001). Differential adhesion of major surface proteins 1a and 1b of the ehrlichial cattle pathogen *Anaplasma marginale* to bovine erythrocytes and tick cells. Int J Parasitol.

[B024] de la Fuente J, Kocan KM, Almazán C, Blouin EF (2007). RNA interference for the study and genetic manipulation of ticks. Trends Parasitol.

[B025] de la Fuente J, Ruybal P, Mtshali MS, Naranjo V, Shuqing L, Mangold AJ (2007). Analysis of world strains of *Anaplasma marginale* using major surface protein 1a repeat sequences. Vet Microbiol.

[B026] Esteves E, Bastos CV, Zivkovic Z, de la Fuente J, Kocan K, Blouin E (2009). Propagation of a Brazilian isolate of *Anaplasma marginale* with appendage in a tick cell line (BME26) derived from *Rhipicephalus* (*Boophilus*) *microplus.*. Vet Parasitol.

[B027] Finkel SE (2006). Long-term survival during stationary phase: evolution and the GASP phenotype. Nat Rev Microbiol.

[B028] Hidalgo RJ, Jones EW, Brown JE, Ainsworth AJ (1989). *Anaplasma marginale* in tick cell culture. Am J Vet Res.

[B029] Hove P, Madesh S, Nair A, Jaworski D, Liu H, Ferm J (2022). Targeted mutagenesis in *Anaplasma marginale* to define virulence and vaccine development against bovine anaplasmosis. PLoS Pathog.

[B030] Husin NA, Khoo JJ, Zulkifli MMS, Bell-Sakyi L, AbuBakar S (2021). Replication Kinetics of *Rickettsia raoultii* in Tick Cell Lines. Microorganisms.

[B031] Kalil SP, da Rosa RD, Capelli-Peixoto J, Pohl PC, de Oliveira PL, Fogaça AC (2017). Immune-related redox metabolism of embryonic cells of the tick *Rhipicephalus microplus* (BME26) in response to infection with *Anaplasma marginale.*. Parasit Vectors.

[B032] Kessler RH (2001). Considerações sobre a transmissão de *Anaplasma marginale.*. Pesq Vet Bras.

[B033] Klumpp S, Zhang Z, Hwa T (2009). Growth rate-dependent global effects on gene expression in bacteria. Cell.

[B034] Kocan KM, de la Fuente J, Blouin EF, Coetzee JF, Ewing SA (2010). The natural history of *Anaplasma marginale.*. Vet Parasitol.

[B035] Kocan KM, de la Fuente J, Blouin EF, Garcia-Garcia JC (2004). *Anaplasma marginale* (Rickettsiales: Anaplasmataceae): recent advances in defining host-pathogen adaptations of a tick-borne rickettsia. Parasitology.

[B036] Kocan KM, de la Fuente J, Guglielmone AA, Meléndez RD (2003). Antigens and alternatives for control of *Anaplasma marginale* infection in cattle. Clin Microbiol Rev.

[B037] Kocan KM, Stiller D, Goff WL, Claypool PL, Eduards W, Ewing SA (1992). Development of *Anaplasma marginale* in male *Dermacentor andersoni* transferred from parasitemic to susceptible cattle. Am J Vet Res.

[B038] Kocan KM, Zivkovic Z, Blouin EF, Naranjo V, Almazán C, Mitra R (2009). Silencing of genes involved in *Anaplasma marginale*-tick interactions affects the pathogen developmental cycle in *Dermacentor variabilis.*. BMC Dev Biol.

[B039] Lima-Duarte L, Camargo JV, Castro-Santiago AC, Machado RZ, André MR, Cabral-de-Mello DC (2021). Establishment and characterization of a cell line (RBME-6) of *Rhipicephalus* (*Boophilus*) *microplus* from Brazil. Ticks Tick Borne Dis.

[B040] Madigan MT, Bender KS, Buckley DH, Slattley M, Stahl DA (2019). Brock biology of microorganisms..

[B041] Munderloh UG, Blouin EF, Kocan KM, Ge NL, Edwards WL, Kurtti TJ (1996). Establishment of the tick (Acari: Ixodidae)-borne cattle pathogen *Anaplasma marginale* (Rickettsiales: Anaplasmataceae) in tick cell culture. J Med Entomol.

[B042] Munderloh UG, Jauron SD, Fingerle V, Leitritz L, Hayes SF, Hautman JM (1999). Invasion and intracellular development of the human granulocytic ehrlichiosis agent in tick cell culture. J Clin Microbiol.

[B043] Munderloh UG, Kurtti TJ (1989). Formulation of medium for tick cell culture. Exp Appl Acarol.

[B044] Munderloh UG, Liu Y, Wang M, Chen C, Kurtti TJ (1994). Establishment, maintenance and description of cell lines from the tick *Ixodes scapularis.*. J Parasitol.

[B045] Munderloh UG, Lynch MJ, Herron MJ, Palmer AT, Kurtti TJ, Nelson RD (2004). Infection of endothelial cells with *Anaplasma marginale* and *A. phagocytophilum.*. Vet Microbiol.

[B046] Passos LMF (2012). *In vitro* cultivation of *Anaplasma marginale* and *A. phagocytophilum* in tick cell lines: a review. Rev Bras Parasitol Vet.

[B047] Richards J, Sundermeier T, Svetlanov A, Karzai AW (2008). Quality control of bacterial mRNA decoding and decay. Biochim Biophys Acta.

[B048] Rodríguez SD, García Ortiz MA, Hernández Salgado G, Santos Cerda NA, Aboytes Torre R, Cantó Alarcón GJ (2000). *Anaplasma marginale* inactivated vaccine: dose titration against a homologous challenge. Comp Immunol Microbiol Infect Dis.

[B049] Rolfe MD, Rice CJ, Lucchini S, Pin C, Thompson A, Cameron ADS (2012). Lag phase is a distinct growth phase that prepares bacteria for exponential growth and involves transient metal accumulation. J Bacteriol.

[B050] Salinas-Estrella E, Amaro-Estrada I, Cobaxin-Cárdenas ME, Preciado de la Torre JF, Rodríguez SD (2022). Bovine Anaplasmosis: will there ever be an almighty effective vaccine?. Front Vet Sci.

[B051] Silaghi C, Kauffmann M, Passos LMF, Pfister K, Zweygarth E (2011). Isolation, propagation and preliminary characterisation of *Anaplasma phagocytophilum* from roe deer (*Capreolus capreolus*) in the tick cell line IDE8. Ticks Tick Borne Dis.

[B052] Solyman MSM, Ujczo J, Brayton KA, Shaw DK, Schneider DA, Noh SM (2022). Iron Reduction in *Dermacentor andersoni* Tick Cells Inhibits *Anaplasma marginale* Replication. Int J Mol Sci.

[B053] Varma MGR, Pudney M, Leake CJ (1975). The establishment of three cell lines from the tick *Rhipicephalus appendiculatus* (Acari: Ixodidae) and their infection with some arboviruses. J Med Entomol.

[B054] Villar M, Ayllón N, Alberdi P, Moreno A, Moreno M, Tobes R (2015). Integrated metabolomics, transcriptomics and proteomics identifies metabolic pathways affected by *Anaplasma phagocytophilum* infection in tick cells. Mol Cell Proteomics.

[B055] Villar M, Ayllón N, Busby AT, Galindo RC, Blouin EF, Kocan KM (2010). Expression of heat shock and other stress response proteins in ticks and cultured tick cells in response to *Anaplasma* spp. infection and heat shock. Int J Proteomics.

[B056] Woldehiwet Z, Horrocks BK, Scaife H, Ross G, Munderloh UG, Bown K (2002). Cultivation of an ovine strain of *Ehrlichia phagocytophila* in tick cell cultures. J Comp Pathol.

[B057] Zhang Y, Zheng J (2020). Bioinformatics of metalloproteins and metalloproteomes. Molecules.

[B058] Zweygarth E, Josemans AI, Spickett AM, Steyn HC, Putterill J, Troskie PC (2006). *In vitro* cultivation of a south African isolate of an *Anaplasma* sp. in tick cell cultures. Onderstepoort J Vet Res.

